# ERK Activity Dynamics during Zebrafish Embryonic Development

**DOI:** 10.3390/ijms20010109

**Published:** 2018-12-28

**Authors:** Kah-Loon Wong, Ryutaro Akiyama, Yasumasa Bessho, Takaaki Matsui

**Affiliations:** Gene Regulation Research, Division of Biological Science, Graduate School of Science and Technology, Nara Institute of Science and Technology, Nara 630-0101, Japan; blckahloon@gmail.com (K.-L.W.); r-akiyama@bs.naist.jp (R.A.); ybessho@bs.naist.jp (Y.B.)

**Keywords:** mitogen-activated protein kinase (MAPK), biosensor, signal activity, vertebrate development

## Abstract

During vertebrate development, extracellular signal-regulated kinase (ERK) is activated by growth factors such as fibroblast growth factor (FGF), and it regulates the formation of tissues/organs including eyes, brains, somites, limbs, and inner ears. However, an experimental system to monitor ERK activity dynamics in the entire body of the vertebrate embryo is lacking. We recently studied ERK activity dynamics in the pre-somitic mesoderm of living zebrafish embryos injected with mRNAs encoding a Förster resonance energy transfer (FRET)-based ERK biosensor. In this study, transgenic zebrafish stably and ubiquitously expressing the ERK biosensor were generated to monitor ERK activity dynamics throughout embryonic development. The system allowed the identification of ERK activation domains in embryos from the late blastula to the late segmentation stage, consistent with immunostaining patterns obtained using anti-phosphorylated ERK antibody. A spatiotemporal map of ERK activity in the entire body during zebrafish embryogenesis was generated, and previously unidentified activation dynamics and ERK domains were identified. The proposed system is the first reported method to monitor ERK activity dynamics during vertebrate embryogenesis, providing insight into the role of ERK activity in normal and abnormal development in living vertebrate embryos.

## 1. Introduction

Extracellular signal-regulated kinase (ERK) mitogen-activated protein kinase is a key signaling mediator of the Ras-Raf-MEK-ERK cascade and is required for a wide range of cellular responses including mitosis, differentiation, survival, migration, and apoptosis during vertebrate embryonic development [1,2,3]. The ERK signaling cascade is activated by extracellular signaling molecules such as fibroblast growth factor (FGF), epidermal growth factor, and vascular endothelial growth factor, which bind to specific receptors to activate the ERK cascade [1]. ERK is phosphorylated and activated by MEK, becoming phosphorylated ERK (pERK) [4]. pERK phosphorylates serine and/or threonine residues of substrate proteins [5]. More than 200 pERK substrates have been identified to date, which may explain the diverse biological functions of ERK signaling [6,7].

ERK activation domains in vertebrate embryos from mouse, chick, frog, and zebrafish were identified by immunohistochemistry using an anti-pERK antibody, which suggested that ERK activation occurs in a spatiotemporal manner [8,9,10,11,12]. To visualize such spatiotemporal dynamics of ERK activity in vertebrates, transgenic mice carrying Förster resonance energy transfer (FRET)-based ERK biosensors were generated using the Tol2 transposase system, which enabled monitoring of ERK activity dynamics in specific embryo organs at postnatal stages [13]. However, the temporal resolution of static analyses (e.g., pERK immunostaining) is low. In addition, mouse embryogenesis occurs in utero and the mouse embryo is large; therefore, a high-resolution spatiotemporal map of ERK activity dynamics covering the entire body of the embryo throughout vertebrate development has not been achieved to date.

To generate a spatiotemporal map of ERK activity dynamics in vertebrate embryos, a good model organism that allows time-lapse analyses in the entire body of the embryo is important. Zebrafish (*Danio rerio*) is the ideal model because zebrafish embryos develop ex utero, have a small and transparent body, and share almost all developmental events/mechanisms with mammals. We recently injected mRNAs encoding the FRET-based ERK biosensor into zebrafish embryos. Although FRET signals from the ERK biosensor differed between regions within individual embryos and among embryos because of technical variations of mRNA injection, we selected optimal samples to visualize ERK activity dynamics within living zebrafish embryos and elucidated the role of ERK activity dynamics in somite segmentation [14]. Stable and ubiquitous expression of the ERK biosensor in zebrafish embryos would provide a powerful tool to observe ERK activity dynamics in the entire body of the vertebrate embryo. In this study, we generated a transgenic zebrafish line ubiquitously expressing the ERK biosensor termed *Tg[ef1α:ERK biosensor-nes]* (*Teen*). This system enabled the observation of ERK activity dynamics in the entire body of zebrafish embryos at the blastula, gastrula, and segmentation stages, during which many key developmental events occur. In addition, we generated a spatiotemporal map of ERK activity in zebrafish embryos at a higher resolution than that of static analyses.

## 2. Results and Discussion

### 2.1. Teen is Useful to Monitor ERK Activity in Living Zebrafish Embryos

**Figure 1:**
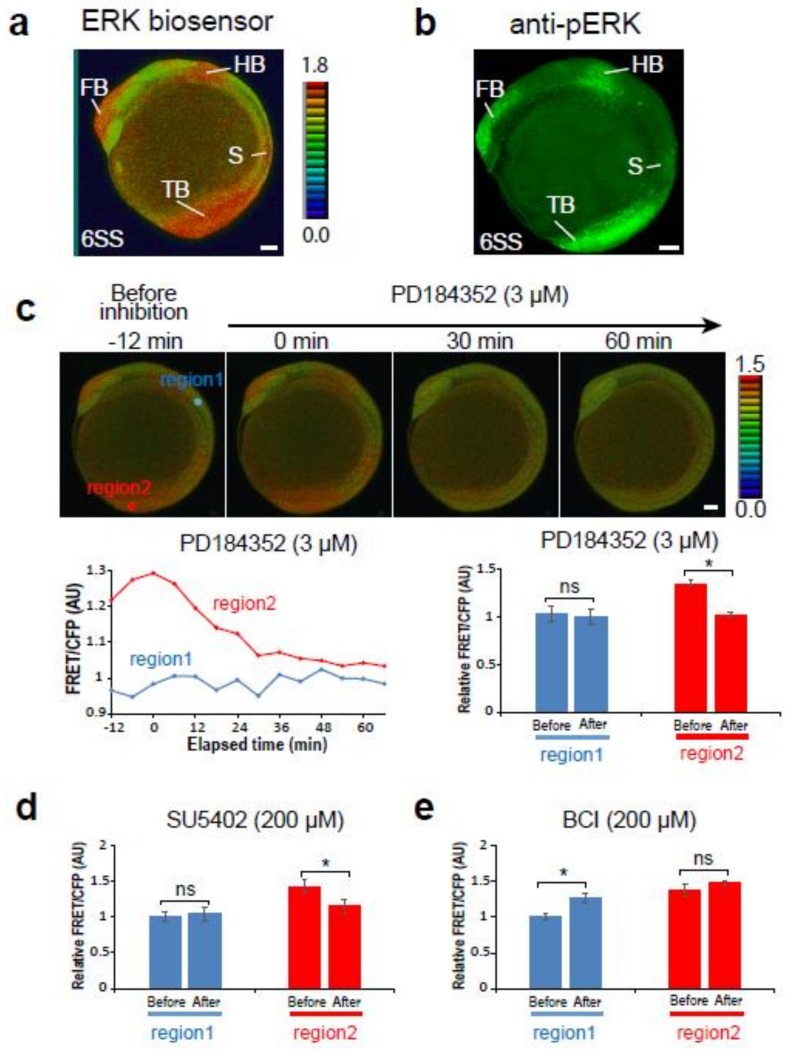
The *Teen* allowed monitoring of extracellular signal-regulated kinase (ERK) activity in living zebrafish embryos. (**a, b**) ERK activity detected by the ERK biosensor (8-color heat map) (**a**) or an anti-pERK antibody (green); (**b**) lateral view. Scale bar, 50 µm. FB, forebrain; HB, hindbrain; S, somite; TB, tail bud. (**c**–**e**) Changes of ERK activity in 6-somite stage *Teen* embryos treated with the MEK inhibitor (3 µM PD184352, *n* = 3) (**c**), the Fgf receptor inhibitor (200 µM SU5402, *n* = 3) (**d**), or the ERK phosphatase inhibitor (200 µM BCI, *n* = 3) (**e**). Region 1, a region between the hindbrain and first somite; region 2, tail bud. Before, 0 min; after, 60 min. * *p < 0.05*; ns, not significant; error bars, standard deviation.

To monitor the spatiotemporal dynamics of ERK activity in the entire body of living zebrafish embryos throughout development, we generated a transgenic zebrafish line termed *Teen* (see Materials and Methods). In *Teen* embryos at the 6-somite stage (12 hours post-fertilization, hpf), cyan fluorescent protein (CFP) signals were detected in the entire body of the embryo, indicating that the ERK biosensor was expressed ubiquitously in the embryo. The signal intensity of the FRET/CFP ratio differed throughout the embryo, and a high FRET/CFP ratio was detected in the forebrain, midbrain-hindbrain boundary, caudal hindbrain, newly formed somites, and tail bud (Figure 1a). This pattern was consistent with the distribution of pERK (active form of ERK) determined by immunostaining (Figure 1b) and the patterns of ERK activity, as shown in embryos injected with mRNAs encoding the ERK biosensor with a nuclear localization signal [14]. These similarities suggested that ERK activity can be monitored in *Teen* embryos.

To confirm the efficacy of the biosensor, *Teen* embryos were treated with PD184352 (MEK inhibitor), SU5402 (inhibitor of the Fgf receptor upstream of the Ras-Raf-MEK-ERK pathway), or BCI (ERK phosphatase inhibitor), and temporal changes in the FRET/CFP ratio were observed. Treatment with the MEK inhibitor or Fgf receptor inhibitor resulted in a gradual decrease in the high FRET/CFP signals in the tail bud of 8SS embryos to basal levels, whereas no changes in the low FRET/CFP signals in the region between the hindbrain and first somite were observed (Figure 1c,d). By contrast, the ERK phosphatase inhibitor increased the FRET/CFP signals in the negative region between the hindbrain and first somite, and not in the tail bud (Figure 1e). These findings were supported by the results of statistical analyses from three independent experiments (Figure 1c–e) and indicated that *Teen* is a suitable system to monitor ERK activity in living zebrafish embryos. 

### 2.2. Generation of a Spatiotemporal Map of ERK Activity during Zebrafish Embryonic Development

To generate a spatiotemporal map of ERK activity during zebrafish embryonic development, time-lapse FRET imaging was performed in *Teen* embryos at several different stages of development, covering most key events of vertebrate embryonic development.

#### 2.2.1. Late Blastula Stage, from Sphere to 50% Epiboly

Cell division, cell rearrangement, and cell fate determination occur in embryos during the late blastula period [15]. In zebrafish, late blastulae of smooth and spherical shape are generated at 4 hpf. Soon after (at approximately 4.3 hpf), yolk cell bulging towards the animal pole generates blastulae with a dome-like structure. The yolk cell bulging leads to the formation of a cup-shaped structure called epiboly (by 4.7 hpf). The cup-like structure expands towards the vegetal pole and gradually covers the yolk cell. Immunostaining for pERK detected ERK activity in the future dorsal region at the sphere stage and in the embryonic margin at the dome stage [8,9]. However, because of the low spatiotemporal resolution of static analyses, the spatial and temporal dynamics of ERK activity are not fully understood. 

**Figure 2:**
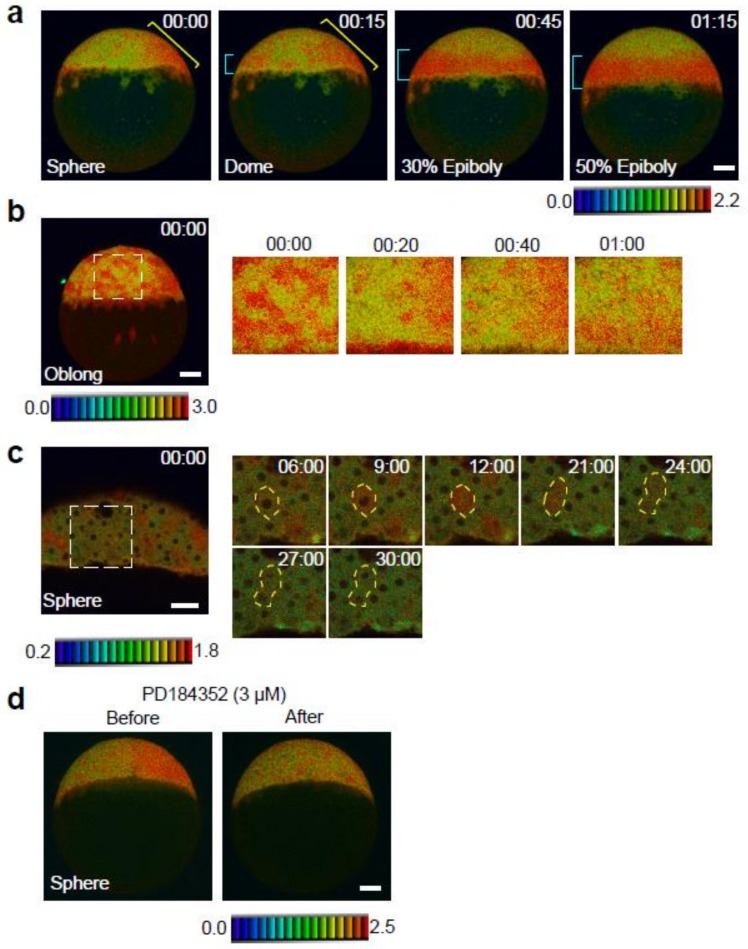
Spatial and temporal map of ERK activity during the late blastula stage. (**a**) ERK activity dynamics from sphere to 50%-epiboly stages. Lateral view, anterior to the top. Blue and yellow brackets indicate the embryonic margin and future dorsal region, respectively. (**b**) ERK activity dynamics in the rectangle (white dotted line) from the left panel. Cells migrate towards the vegetal pole, and changes in ERK activation from high to low (or from low to high) can be seen. (**c**) ERK activation during cell cycle progression. Higher magnification view of the rectangle (white dotted line) in the left panel. Before cell cleavage (M phase), ERK activity increased, whereas it was low in other phases. The yellow dotted line marks the outline of the cell. (**d**) Changes of ERK activity in sphere stage *Teen* embryos treated with the MEK inhibitor (3 µM PD184352, *n* = 6). Before, 0 min; after, 60 min. Scale bar, 100 µm.

In *Teen* embryos at the sphere stage, ERK activity, as indicated by high FRET/CFP signals, was detected in the future dorsal region (Figure 2a), consistent with the results of pERK immunostaining [16]. Time-lapse FRET imaging detected the transition of ERK activity dynamics from the future dorsal region to the embryonic margin for approximately 1 h (from sphere to 50% epiboly; 4–5.5 hpf) at high spatiotemporal resolution (Figure 2a and Supplementary Materials Movie 1). During the transition, ERK activity decreased at the dorsal side of the embryo near the animal pole, remained stable at the dorsal side of the embryo near the embryonic margin, and increased at the embryonic margin on the ventral and medial regions (Figure 2a and Supplementary Materials Movie 1). Time-lapse imaging at higher magnification showed severe cell rearrangement and a change in ERK activation from high to low (or vice versa) during this transition (Figure 2b and Supplementary Materials Movie 2). In addition, fluctuations of ERK activity were detected in small patches of cells (Figure 2c). Careful observation of cell dynamics showed that the ERK biosensor detected ERK activation during cell cycle progression: ERK activity increased before cell cleavage (at the M phase entry), whereas it was low in other phases (Figure 2c and Supplementary Materials Movie 3). This is supported by previous findings that asynchronous cell divisions occur in embryos during the blastula period [17]. However, as the ERK substrate sequence in the ERK biosensor shares a consensus phosphorylation sequence of CDK1 substrates, it is possible that these signals at the M phase entry do not depend on ERK activity, as previously reported [18]. To test the possibility, we inhibited ERK activation by treatment of the MEK inhibitor. Almost all FRET/CFP signals were inhibited by the MEK inhibitor, but patchy signals at the M phase entry remained (Figure 2d, right panel), indicating that the cyclin B/CDK1 complex may phosphorylate the ERK biosensor in the M phase. Therefore, these results suggest that dynamic changes in ERK activity during the late blastula period are mediated by a complex developmental event involving the spatiotemporal regulation of ERK signaling, and/or cell rearrangement. 

#### 2.2.2. Gastrula Stage, from 50%-Epiboly to Bud Stage

During the gastrula period, three primary germ layers (endoderm, ectoderm, and mesoderm) are generated within embryos, whereas epiboly itself continuously proceeds towards the vegetal pole until it covers the entire yolk cell by the end of the gastrula period [15]. In previous studies, immunostaining with an anti-pERK antibody detected pERK signals in the embryonic margin at approximately 50%-epiboly stage (5.5 hpf) and in three domains called the presumptive forebrain, presumptive hindbrain, and tail bud at around the bud stage (10 hpf) [8,9,19,20,21].

**Figure 3:**
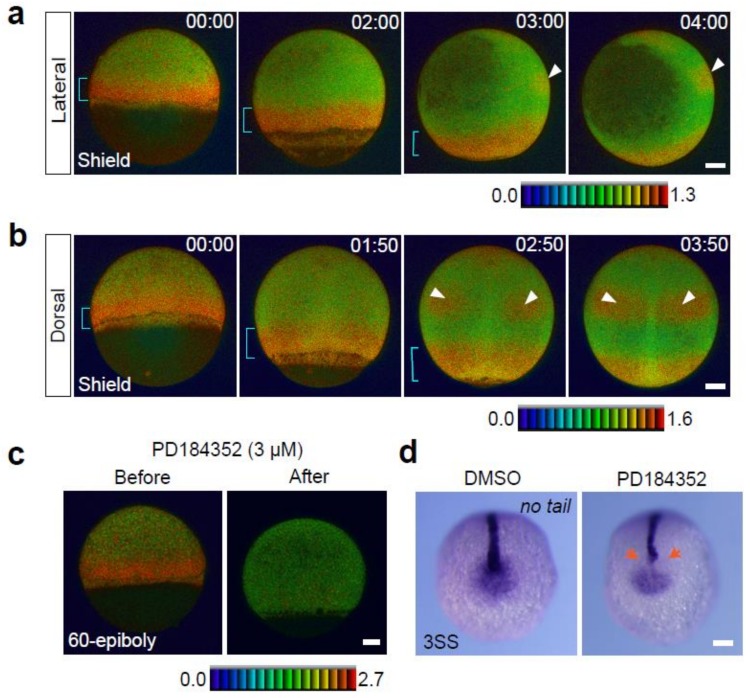
Spatial and temporal map of ERK activity during the gastrula stage. (**a**) Lateral view, anterior to the top. (**b**) Dorsal view, anterior to the top. A blue bracket indicates the embryonic margin. White arrowheads mark the future hindbrain. (**c**) Changes of ERK activity in gastrula stage *Teen* embryos treated with the MEK inhibitor (3 µM PD184352, *n* = 8). Before, 0 min; after, 60 min. (**d**) *no tail* expression in 3SS embryos treated with DMSO (*n* = 21) or PD184352 (*n* = 24) for 2 h (from shield to 75-epiboly stages). Inhibition of ERK during the gastrula stages leads to failures of mesodermal cell patterning in notochord and tail bud. Scale bar, 100 µm.

Our live imaging system detected high ERK activity only in the embryonic margin at the shield stage (6 hpf) (Figure 3a,b, and Supplementary Materials Movies 4 and 5). The domain possessing ERK activity moved towards the vegetal pole during epiboly progression and reached the vegetal pole by bud stage (10 hpf) (Figure 3a,b) in a region termed the tail bud. Inhibition of ERK activation during gastrulation stages resulted in a decrease of ERK activity in the embryonic margin but did not block migration of these cells during epiboly (Figure 3c). Instead, this inhibition led to the failures of patterning of mesodermal cells in the notochord and tail bud (Figure 3d). These results suggest that cells in the tail bud originate from cells at the embryonic margin during the early gastrula period and that ERK activity in these cells is maintained at a high level throughout the gastrula stage (for approximately 5 h) and is required for the following developmental events.

In the early gastrula period (shield stage–75%-epiboly stage, 6–8 hpf), ERK activity was detected only at the embryonic margin (Figure 3a,b). However, after 75% epiboly, low ERK activity was detected in new domains around the animal pole and the animal pole side of the equatorial plane of the embryos (Figure 3, arrowheads). During late gastrula (75%-epiboly–bud stage, 8–10 hpf), ERK activity gradually increased in these regions and was finally detected in regions called the presumptive forebrain and presumptive hindbrain at the bud stage. The mode of appearance of ERK activity suggested that ERK activation occurs in these regions during the late gastrula. This notion was supported by previous findings that *fgf3* and *fgf8a*, which are activators of the Ras-Raf-MEK-ERK signaling cascade, are expressed in these regions at this stage [22,23].

#### 2.2.3. Segmentation Stage, from the 6-somite to the 22-somite Stage 

**Figure 4:**
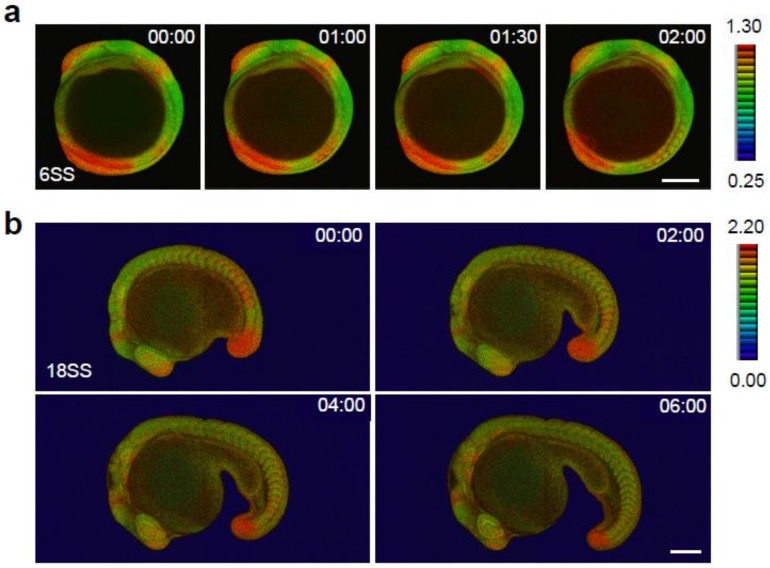
Spatial and temporal map of ERK activity during somitogenesis. (**a**) ERK activity dynamics from 6SS to 10SS stages. Lateral view, anterior to the top. (**b**) ERK activity dynamics from 18SS to 30SS stages. Lateral view, anterior to the left. Scale bar, 200 µm.

**Figure 5:**
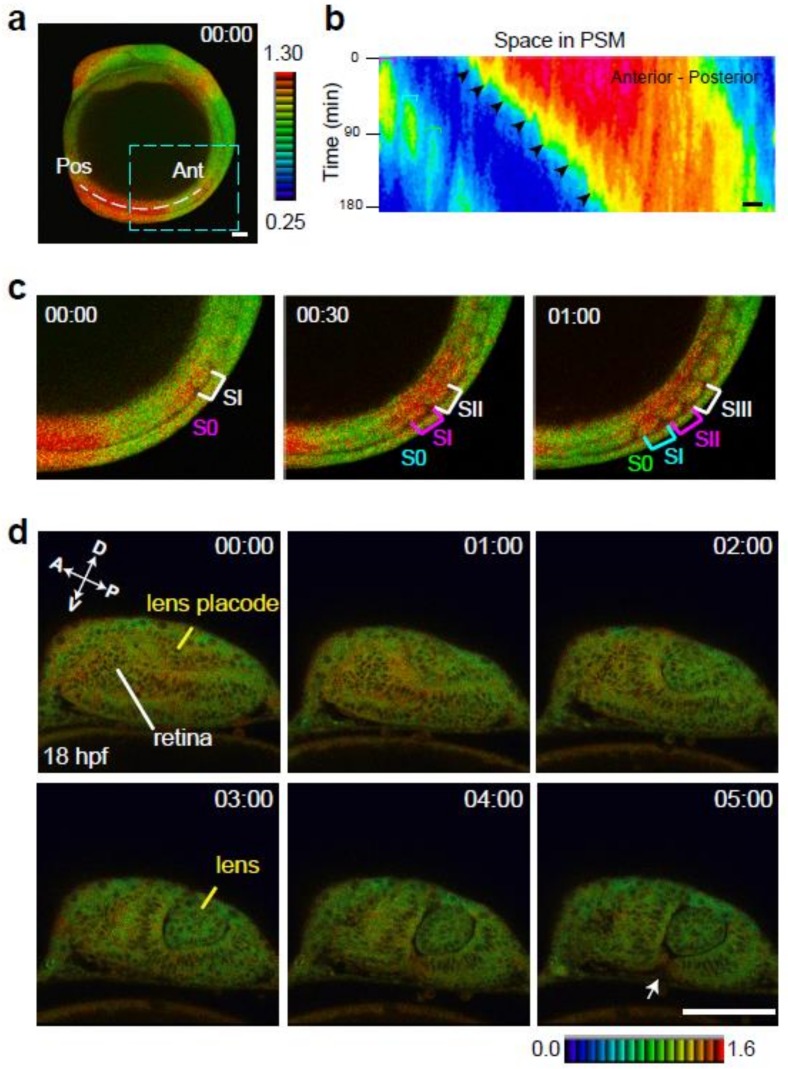
ERK activity dynamics in specific tissues/organs. (**a**) A white dotted line represents the region of interest for kymograph analysis. (**b**) Kymograph of the spatial and temporal dynamics of ERK activity in the presomitic mesoderm at the tail bud. Horizontal and vertical axes indicate the length from the anterior end of the presomitic mesoderm (µm) and time progression (min), respectively. Scale bar, 50 µm. (**c**) Higher magnification of the rectangle (light blue dotted line) in panel **a**. Newly developed somites are indicated by S0. Brackets indicate somite formation. The appearance of S0 (pink) indicates ERK activation within the S0. ERK activation level is high when S0 becomes SI (pink bracket), and the level decreases when SI becomes SII (pink bracket). Scale bar, 100 µm. (**d**) At 18 hpf, ERK activation is observed in the retina and lens placode. During lens formation (18–20 hpf), ERK activation decreases gradually in the lens placode and remains stable in the retina. ERK activity decreases in the anterior side of the retina and is then restricted to the fissure of the retina on the ventral side.

The segmentation stage is characterized by a variety of morphological events. The anterior-posterior and dorsal-ventral axes become obvious. Organ primordia including eye, brain, heart, and gut begin to form. Somite development occurs in association with tail elongation. Heartbeats and body movements appear at the late segmentation stage. Immunostaining for pERK showed that ERK is activated in the forebrain, midbrain-hindbrain boundary, eye, newly formed somite, and tail bud in embryos at several segmentation stages (e.g., 6-, 12-, and 22-somite stages) [8,22,24]. Consistent with previous findings, we detected ERK activation in the forebrain, midbrain-hindbrain boundary, newly formed somites, and tail bud of *Teen* embryos at the 6-somite stage (12 hpf) (Figure 1a and Figure 4a). ERK was activated in the presumptive brain regions and tail bud at the late gastrula stage (Figure 3), and ERK activation in the forebrain, the midbrain-hindbrain boundary, part of the hindbrain, and the tail bud was maintained during late gastrula and segmentation stages (Figure 4 and Supplementary Materials Movies 6 and 7), suggesting the existence of a continuous activation system for ERK in these regions. We previously reported that the stepwise regression of ERK activity occurs in the presomitic mesoderm at the tail bud [14,24], which is required for the pre-pattern formation of somites. Here, a stepwise shift of the ERK activation border was observed in *Teen* embryos (Figure 5a,b, and Supplementary Materials Movie 6).

Taken together, these data indicated that live imaging of ERK activity dynamics using the *Teen* zebrafish reconfirmed the existence of ERK activation domains, which were previously detected by pERK immunostaining, and defined the spatial and temporal dynamics of ERK activity during embryonic development. The data indicate that the *Teen* zebrafish is an efficient and reliable tool to investigate the regulatory mechanism of ERK activity dynamics and the relationship between ERK activity dynamics and the morphological events occurring during vertebrate development. 

### 2.3. Identification of Novel ERK Activity Dynamics and Domains

Time-lapse FRET imaging in *Teen* embryos was effective for identifying novel spatiotemporal changes in ERK activity in specific organs/tissues. The technique enabled the identification of ERK activation dynamics in the eyes, newly formed somites (SI), and developing somites (S0) (Figure 5). In zebrafish, somite formation occurs every 30 min through periodic segmentation of the presomitic mesoderm at the tail bud [24,25,26]. Newly formed somites (new S0) appear every 30 min, and SI and S0 become new SII and SI, respectively. Transient activation of ERK occurred during S0-SI-SII transitions: ERK was newly activated in S0, whereas the activation status was maintained in SI and decreased in SII (Figure 5c and Supplementary Materials Movie 6). Because S0-SI-SII transitions are accompanied by somite generation cycles, cyclic changes of ERK activity were observed every 30 min during these transitions (Figure 5c and Supplementary Materials Movie 6).

At the 18-somite stage (18 hpf), ERK was activated in the lens placode and anterior half of the retina. During the formation of the lens from the placode (18–20 hpf), ERK activity decreased gradually in cells of the lens. In the retina, ERK activity decreased in the anterior side of the retina, whereas it was maintained in cells adjacent to the lens. Thereafter, ERK activation was restricted to the fissure of the retina on the ventral side (Figure 5d and Supplementary Materials Movie 8). These findings regarding changes in ERK activation within specific tissues/organs are novel. It would be of great interest to apply this system to identify additional spatial and temporal ERK activity dynamics, which would provide insight into the regulation of ERK activity in tissues/organs during embryonic development.

### 2.4. A Previously Unidentified Role of ERK Activity in the Tail Bud of Zebrafish Embryos

**Figure 6:**
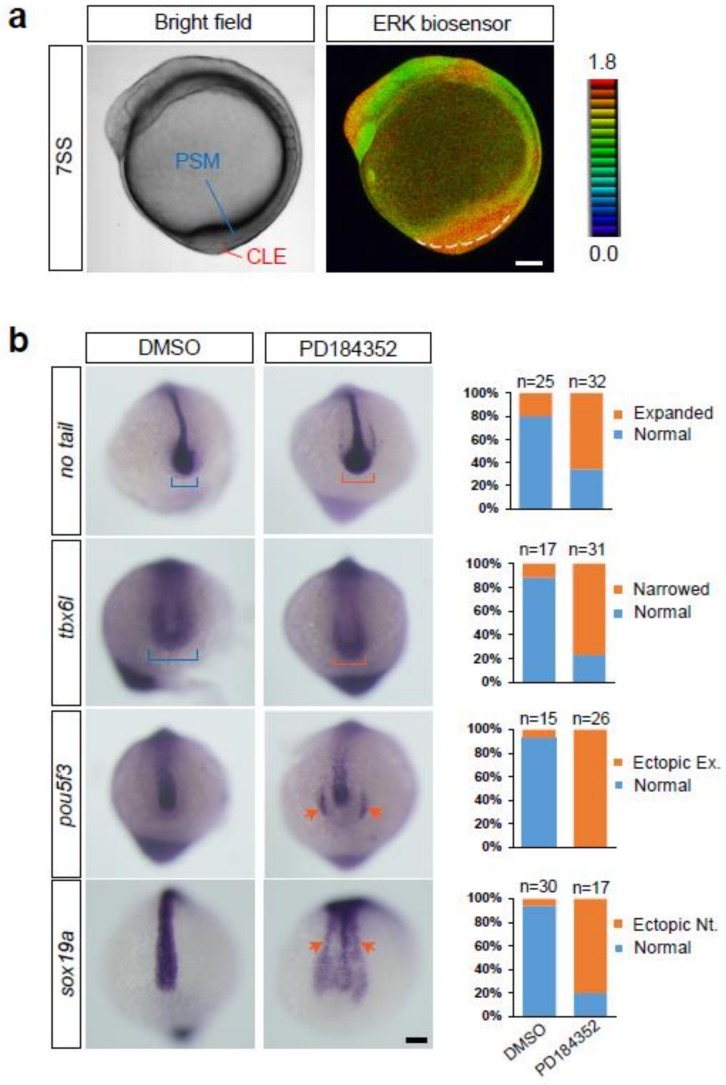
Roles of ERK activity in the tail bud. (**a**) Bright field (left panel) and ERK activity (right panel) of the embryo at the 7-somite stage. PSM, pre-somitic mesoderm; CLE, caudal lateral epiblast. Lateral view, anterior to the top. Scale bar, 100 µm. (**b**) Expression of *no tail*, *tbx6l*, *pou5f3*, and *sox19a* in embryos treated with DMSO (vehicle) or the MEK inhibitor PD184352. In MEK inhibitor-treated embryos, a pool of axial stem cells (caudal region of the neural tube), which is marked by *no tail* expression, expanded (orange bracket, the first row from the top), and the paraxial mesoderm, marked by *tbx6l*, became narrower (orange bracket, second row from the top). In addition, ectopic neural tube formation, as evidenced by expression of *pou5f3* (a marker of neural progenitors; orange arrows, third row from the top) and *sox19a* (orange arrows, fourth row from the top), was frequently induced in MEK inhibitor-treated embryos. Scale bar, 100 µm.

In this study, we briefly surveyed ERK activity dynamics in *Teen* embryos during the late blastula, gastrula, and segmentation stages. Precise detection of ERK activity in specific regions of the embryo at specific stages of development using our system would provide higher resolution data on the spatiotemporal dynamics of ERK activity. Assessment of ERK activity in the tail bud regions at the 6-somite–10-somite stages (12–14 hpf) indicated that ERK is not only activated in the pre-somitic mesoderm, but also in the upper layer of the pre-somitic mesoderm, which includes the posterior section and the caudal region of the neural tube (Figure 6a). Because the anterior limit of ERK activity in the upper layer was similar to that of the pre-somitic mesoderm, we overlooked activation of ERK in the upper layer (i.e., the posterior neural tube and the caudal region of the neural tube) in our previous analyses [14,24].

In the chick and mouse, the caudal region of the neural tube serves as a pool of axial stem cells, which are progenitors of the paraxial mesoderm (pre-somitic mesoderm) and the posterior neural tube. Transcriptional control of the transcription factor *Sox2* (a marker of neural primordial cells) is crucial for the differentiation of mesodermal and neural cells from axial stem cells [27,28]. In the pool of axial stem cells, Wnt signaling activates *Sox2* transcription, whereas BMP signaling suppresses *Sox2* transcription. Thus, *Sox2* expression is maintained at a low level in the pool. In the caudal end of the neural tube, BMP signaling is turned off, whereas Wnt signaling remains active, and *Sox2* expression is upregulated, leading to differentiation into neural cells. However, in the caudal region of the pre-somitic mesoderm, both BMP and Wnt signals decrease and *Sox2* expression is downregulated; the T-box transcription factor Tbx6, which is specifically expressed in the paraxial mesoderm, also inhibits *Sox2* expression, leading to cell fate commitment into a mesodermal fate. Fgf-ERK signaling can activate *Sox2* expression, whereas loss-of-function of Fgf-ERK signaling fails to maintain axial stem cells in the caudal region of the neural tube, leading to a defect of elongation of the body axis. 

In the zebrafish, Fgf signaling is required for paraxial mesoderm induction during somitogenesis [29]. However, whether Fgf-ERK signaling maintains axial stem cells in zebrafish embryos remains to be determined. To reveal the role of Fgf-ERK signaling in the maintenance of axial stem cells in zebrafish, we treated *Teen* embryos with a MEK inhibitor at the bud–10-somite stage (10–14 hpf). MEK inhibition decreased ERK activation to basal levels within 1 h (Figure 1c), leading to shortening of the anterior-posterior axis (100%, *n* = 106); this was consistent with results obtained in chick and mouse embryos [30,31]. However, unexpected results suggested a distinct/alternative role of ERK signaling in regulating the balance of neural and mesodermal fates from axial stem cells (Figure 6b). When ERK activation was suppressed by the MEK inhibitor in zebrafish embryos during the early segmentation period, *no tail* expression was normally detected in the notochord, and it expanded to the caudal region of the neural tube (66%; *n* = 32), suggesting an increase in stem cell populations, which differs from the phenotypes in mouse and chick embryos. Furthermore, the paraxial mesoderm marked by *tbx6l* (a zebrafish counterpart of mouse *Tbx6*) became narrower (77%; *n* = 31), and ectopic neural tube formation, as indicated by the expression of *pou5f3* (a marker of neural progenitors) and *sox19a* (a zebrafish counterpart of mouse *Sox2*) [32], was frequently induced in MEK inhibitor-treated embryos (92%, *n* = 26, and 80%, *n* = 30, respectively). These results suggest that ERK activity at the posterior section of the neural tube, the caudal region of the neural tube, and the paraxial mesoderm is required for maintaining the proper balance of stem cell, neural, and mesodermal fates in zebrafish.

In the current study, we generated a transgenic zebrafish termed *Teen* and performed time-lapse FRET imaging during zebrafish development, resulting in the generation of a spatiotemporal map of ERK activity dynamics in zebrafish during the late blastula, gastrula, and segmentation stages. In addition, we found previously unidentified ERK activation dynamics and domains using *Teen* zebrafish, which will be useful to investigate the roles of ERK and/or ERK dynamics during vertebrate embryonic development in the near future. The proposed system is the first reported method for monitoring ERK activity dynamics during vertebrate embryogenesis and provides insight into the roles of ERK activity in normal development and developmental disorders within living vertebrate embryos.

## 3. Materials and Methods

### 3.1. Experimental Model

Wild-type zebrafish were used in this study. All zebrafish experiments were performed with the approval of the Animal Studies Committee of Nara Institute of Science and Technology (Approval code: 1317, 1 April 2013).

### 3.2. Generation of Teen Zebrafish Line with the Tol2 Transposon System

The ERK biosensor-nes EKAREV comprises a yellow fluorescent protein for energy transfer (Ypet), a WW domain, an EV linker, an ERK substrate, an enhanced CFP, and a nuclear export signal (nes) [33]. When ERK phosphorylates the ERK substrate, the WW domain binds to the ERK substrate, thereby bringing CFP closer to Ypet and inducing FRET from CFP to Ypet. The *pT2A-ef1α:ERK biosensor-nes* was generated by insertion of the EKREV DNA fragment between the ef1α promoter and SV40 polyA of the pT2AL200R150G plasmid [34], which carries minimal Tol2 elements required for transposition.

The *Teen* zebrafish line was generated with the Tol2 transposon system [35]. Wild-type zebrafish embryos at the one-cell stage were co-injected with the plasmid DNA of *pT2A-ef1α:ERK biosensor-nes* (50 pg) and transposase mRNA (50 pg). The CFP and Ypet fluorescence of injected embryos were detected at the segmentation stage (12–24 hpf), and possible carriers of the plasmid DNA were selected and raised to adulthood. Germ transmitting founder fish of the ERK biosensor (F0) were selected by mating the possible carriers with wild-type fish. An F0 founder fish was crossbred with wild-type fish, and the offspring of the crossbreed were raised and kept as F1. Five generations (F1–F5) were generated from the F0 founder, and no alterations in the CFP and Ypet signals of the ERK biosensor were detected. In addition, no abnormal developmental phenotype was observed in the *Teen* embryos.

### 3.3. FRET Microscopy

FRET signals were observed by LSM710 confocal microscopy (Zeiss) as recently reported [14]. *Teen* embryos were mounted in 1% low-melting agarose and excited with a 440 nm laser, and signals were detected with the lambda scanning mode at 28.5°C. The linear unmixing mode was used to separate CFP and Ypet signals from the initial spectra data. MetaMorph software (Molecular Devices) was used to relate the Ypet image to the CFP image to generate FRET/CFP ratio images. The FRET/CFP ratio images were presented in the intensity-modulated display mode, which shows differences in the FRET/CFP ratio as a 16-color heat map from red (high) to blue (low).

### 3.4. Time-lapse FRET Imaging

To determine whether *Teen* embryos could be used to monitor ERK activity, *Teen* embryos at the 6-somite stage were observed for 60 or 75 min, at 3 or 5 min intervals at 28.5°C. At time = 0 min, the embryos were treated with SU5402 (200 μM), PD184352 (3.0 μM), or BCI (200 μM BCI). 

To observe the dynamic changes in ERK activity throughout zebrafish development, Z-stack images (8–10 planes acquired at 5 or 10 μm intervals) of *Teen* embryos at different stages were obtained at 5 or 10 min intervals at 28.5°C. At least three independent experiments were performed, and representative images are shown in the manuscript.

### 3.5. Whole-mount In Situ Hybridization and Immunohistochemistry

Whole-mount in situ hybridization and immunohistochemistry were performed as described previously [24,36]. cDNA fragments encoding *no tail*, *tbx6l*, *pou5f3*, and *sox19a* were used as templates for antisense probes. An anti-pERK antibody (Sigma-Aldrich, St. Louis, MI, USA, M8159) was used.

### 3.6. Statistical analysis

Differences between means were analyzed by one-tailed Student’s t-test. The results of the t-tests were considered significant when *p < 0.05*.

## Supplementary Materials

Supplementary materials can be found at http://www.mdpi.com/1422-0067/20/1/109/s1.

## Abbreviations

ERK	Extracellular signal-regulated kinase
FRET	Förster resonance energy transfer
CFP	Cyan fluorescent protein
nes	Nuclear export signal
*Teen*	*Tg[ef1α:ERK biosensor-nes]*
