# Supplementary material for: ERK Activity Dynamics during Zebrafish Embryonic Development
Source: Int J Mol Sci. 2018 Dec 28;20(1):109. doi: 10.3390/ijms20010109 (PMC6337290; doi:10.3390/ijms20010109)
Supplement: Supplementary file 1 [file ijms-20-00109-s001.zip › ijms-403023 supp/Movie Captions.docx]

Movie 1. ERK activity during late blastula

ERK activity (16-color heat map) was observed at 5 min intervals. Lateral view.

Movie 2. ERK activity dynamics during late blastula

ERK activity (16-color heat map) was observed at 10 min intervals. Lateral view. Cells migrate towards the vegetal pole, and changes in ERK activity from high (red) to low (green) (or from low to high) are detected.

Movie 3. ERK activity dynamics during cell division

ERK activity (16-color heat map) was observed at 3 min intervals. Lateral view. Before cell cleavage (M phase), ERK activity became higher (red), whereas activity was low (green) in other phases.

Movie 4. ERK activity during the gastrula stage

ERK activity (16-color heat map) was observed at 5 min intervals. Lateral view.

Movie 5. ERK activity during the gastrula stage

ERK activity (16-color heat map) was observed at 5 min intervals. Dorsal view.

Movie 6. ERK activity during early somitogenesis

ERK activity (16-color heat map) was observed at 10 min intervals. Lateral view.

Movie 7. ERK activity during late somitogenesis

ERK activity (16-color heat map) was observed at 10 min intervals. Lateral view.

Movie 8. ERK activity dynamics during eye formation

ERK activity (16-color heat map) was observed at 5 min intervals. Lateral view.
